# Supplementary material for: Comparative analysis of cerebrospinal fluid neurofilament medium, light and heavy chain in neurodegenerative diseases using an in-house assay for the detection of neurofilament medium chain
Source: eBioMedicine. 2025 Sep 19;120:105930. doi: 10.1016/j.ebiom.2025.105930 (PMC12481134; doi:10.1016/j.ebiom.2025.105930)
Supplement: Supplementary Material [file mmc1.docx]

**Comparative analysis of cerebrospinal fluid neurofilament medium, light and heavy chain in neurodegenerative diseases using an in-house assay for the detection of neurofilament medium chain**

Badrieh Fazeli^1^, Sara Botzenhardt^1^, Franziska Bachhuber^1^, Paula Klassen ^1,2^, Veronika Klose ^1,2^, Johannes Dorst^1^, Maximilian Wiesenfarth^1^, Zeljko Uzelac^1^, Sarah Jesse^1^, David Brenner^1^, Sarah Anderl-Straub^1^, Albert C. Ludolph^1^, Markus Otto^3^, Jochen Weishaupt^1^, Hayrettin Tumani^1^, Steffen Halbgebauer^1,2*^

**Affiliations**

^1^ Department of Neurology, Ulm University Hospital, Oberer Eselsberg 45, 89081 Ulm, Germany

^2^ German Centre for Neurodegenerative Diseases (DZNE e.V.), Oberer Eselsberg 45, 89081 Ulm, Germany

^3^ Department of Neurology, University Hospital Halle, Ernst-Grube Strasse 40, 06120 Halle (Saale), Germany

**Corresponding author**

*Steffen Halbgebauer**,** Department of Neurology, Ulm University Hospital, Oberer Eselsberg 45, 89081 Ulm, Germany. Email: [steffen.halbgebauer@uni-ulm.de](mailto:steffen.halbgebauer@uni-ulm.de), Tel. +4973150063112, Fax: +497315001263112

**Table S1. Diagnosis and neurofilament concentrations of the CTRL.DD group**

| Diagnosis | N | female/male | Age (year) | CSF NfM (pg/mL) | CSF NfL (pg/mL) | CSF NfH (pg/mL) |
| --- | --- | --- | --- | --- | --- | --- |
| Benign fasciculations | 6 | 1/5 | 49 (43-57) | 698 (465-1747) | 598 (324-795) | 873(645-1181) |
| brachial plexus injury | 3 | 1/2 | 57 (45-60) | 796 (687-1019) | 441 (355-463) | 751 (602-793) |
| HSP | 2 | 1/1 | 59 (56-62) | 1174 (1049-1298) | 696 (653-739) | 874 (770-979) |
| IBM | 3 | 2/1 | 71 (70-77) | 4353 (1587-4577) | 1092 (867-1516) | 1503 (1362-1533) |
| Multifocal motor neuropathy | 2 | 0/2 | 66 (66-67) | 1078 (879-1277) | 677 (582-772) | 1211 (1205-1216) |
| Myopathy | 7 | 3/4 | 54 (40-65) | 1109 (677-2721) | 482 (463-1091) | 829 (708-1423) |
| PNP | 13 | 3/10 | 71 (53-75) | 1819 (687-3622) | 801 (525-1343) | 1285 (681-1986) |
| Bilateral L5 radiculitis | 1 | 0/1 | 66 | 342 | 578 | 1010 |
| Cervical and lumbar disk herniation | 1 | 0/1 | 52 | 555 | 506 | 531 |
| Cervical disk prolapse | 1 | 1/0 | 56 | 659 | 424 | 891 |
| Fibromyalgia | 1 | 1/0 | 59 | 1106 | 501 | 714 |
| Inclusion body myositis | 1 | 0/1 | 43 | 503 | 305 | 521 |
| Mononeuritis multiplex | 1 | 0/1 | 79 | 2066 | 868 | 1275 |
| MS | 1 | 0/1 | 50 | 875 | 329 | 814 |
| Myelitis | 1 | 0/1 | 50 | 810 | 587 | 719 |
| Myositis | 1 | 1/0 | 78 | 1781 | 1176 | 978 |
| post-polio syndrome | 1 | 0/1 | 63 | 1304 | 671 | 1003 |
| SMA | 1 | 0/1 | 54 | 856 | 433 | 639 |
| Somatic symptom disorder | 1 | 1/0 | 38 | 165 | 231 | 335 |

Data is reported as median (Interquartile range).

CTRL.DD, control patients with initial diagnostic suspicion of ALS but final diagnosis of different condition; CSF, cerebrospinal fluid; HSP, hereditary spastic paraplegia; IBM, Inclusion body myopathy; MS, multiple sclerosis; NfH, neurofilament heavy chain; NfL, neurofilament light chain; NfM, neurofilament medium chain; PNP, polyneuropathy; SMA, spinal muscular atrophy.

**Cross reaction with NfL and NfH**

Given the structural similarities between the neurofilament subunits, we investigated the potential for cross-reactivity between NfL, NfM and NfH. To this end, we tested the affinity of all antibodies for recombinant NfM (Cat. # TP324475), NfL (Cat. # ab224840) and NfH (Cat. # TP313487) using an indirect ELISA where the recombinant protein was coated to the bottom of the wells (data are not shown). Results are presented for the two antibodies used in the final assay (capture antibody, Cat. # CF506794; detector antibody, Cat. #NBP2-72977). To confirm the presence of recombinant NfL and NfH, specific NfL and NfH antibodies were included in the measurements (NfL antibody, Cat. # 130400; NfH antibody, Cat. # 18934-1-AP).

In the indirect ELISA the capture and detector NfM antibodies generated strong signals for NfM and signals for NfL and NfH at the blank level. Only the capture antibody also showed a bottom-line signal for NfH in the indirect ELISA. However, when we assessed the cross-reactivity of the final antibody combination by using all three recombinant proteins as samples in the developed sandwich ELISA, zero cross reaction with NfL and NfH was detected (Figure S1).

**Figure S1. Assessment of cross reactivity with other neurofilament proteins.**

Box plots showing the signals for the three recombinant neurofilament proteins (NfL, NfM, and NfH), produced by antibodies in indirect and sandwich ELISAs. The 13-0400 and 18934-1-AP are used as positive controls for NfL and NfH recombinant proteins. Ab, antibody; NfH, neurofilament heavy chain; NfL, neurofilament light chain; NfM, neurofilament medium chain; Rec, recombinant protein.

**Quantification of CSF NfL and NfH**

NfL and NfH concentrations in CSF were measured using commercial microfluidic Ella kits (Bio-techne, Minneapolis, USA). Frozen CSF samples were thawed at room temperature and centrifuged at 10,000g for 5 min. Sample diluents were equilibrated to room temperature, and CSF samples were diluted 1:4 using sample diluent (SD)13 for NfL and SD06 for NfH. A volume of 50 µL of each diluted sample was loaded into the cartridge wells, followed by the addition of wash buffer, according to the manufacturer’s instructions. The cartridges were then processed using the Ella instrument to quantify biomarker levels. Two quality control (QC) samples were run in duplicate on each plate.

**Assay performance validation**


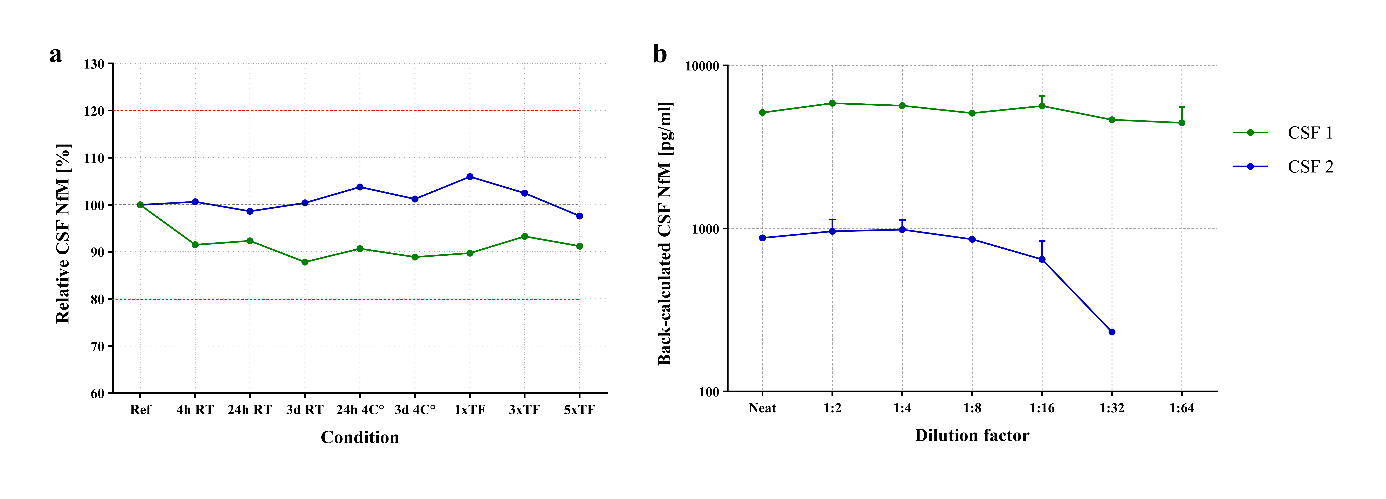
Stability assessments were carried out for two CSF samples. The obtained data revealed that NfM concentrations exhibited less than 20% variation when stored for up to three days at either 4 °C or room temperature. Additionally, changes in CSF NfM concentrations remained within an acceptable range of ±20% after undergoing five freeze-thaw cycles (Figure S2a). Dilution-adjusted concentrations of two CSF samples in the parallelism test were plotted (Figure S2b), and a 1:2 dilution was chosen as the minimum required dilution.

**Figure S2. NfM stability and parallelism assessment in CSF.**

(a) The stability of NfM in CSF was determined by comparing the relative content of NfM in two CSF samples after storage at room temperature or 4 °C and multiple freeze-thaw cycles, in comparison to the reference samples. Variations were found to be less than 20 %. CSF NfM remained stable after undergoing up to five freeze-thaw cycles and storage at room temperature or 4°C up to three days. (b) Back-calculated NfM concentrations within a serial dilution of two CSF samples. CSF, cerebrospinal fluid; NfM, neurofilament medium chain; TF, freeze-thaw cycle.

|  | Control | | | | ALS | | AD | | FTD | | | | | | | | LBD | | | | |
| --- | --- | --- | --- | --- | --- | --- | --- | --- | --- | --- | --- | --- | --- | --- | --- | --- | --- | --- | --- | --- | --- |
| Subgroups | CTRL | | CTRL.DD | | - | | - | | bvFTD | | lvPPA | | nfvPPA | | svPPA | | PD | | PDD | | |
| Sex* | Male | Female | Male | Female | Male | Female | Male | Female | Male | Female | Male | Female | Male | Female | Male | Female | Male | Female | Male | Female |  |
| N | 25 | 26 | 33 | 15 | 47 | 44 | 23 | 36 | 13 | 4 | 4 | 3 | 4 | 3 | 5 | 2 | 8 | 6 | 3 | 1 |  |
| Age at LP [year] | 56 (49-63) | 61 (53-67) | 60 (50-71) | 56 (41-70) | 68(58-77) | 60(54-71) | 61(60-65) | 62(57-65) | 63(56-66) | 67(60-78) | 72(69-74) | 62(56-72) | 75(61-79) | 68(60-78) | 68(59-76) | 58(57-59) | 68(60-72) | 65(53-69) | 75(70-76) | 77 |  |
| CSF NfM [pg/mL] | 772 (568-1274) | 944 (542-1844) | 1019 (701-2056) | 1106 (497-1819) | 11195 (5656-19503) | 16655 (9507-26101) | 3889 (3035-5735) | 5660 (2912-7911) | 2908 (1432-19516) | 10988 (2141-19691) | 6434 (4916-7265) | 2806 (1828-3227) | 11856 (9437-20126) | 14634 (4206-35280) | 10517 (8251-15644) | 13824 (10854-16793) | 1541 (814-3155) | 1654 (932-7149) | 2704 (1259-4559) | 3671 |  |
| CSF NfL [pg/mL] | 563 (471-639) | 569 (377-851) | 597 (479-882) | 501 (379-1092) | 5348 (3444-9334) | 6432 (4564-9181) | 1236 (952-1532) | 1326 (1069-1751) | 1504 (1006-6350) | 2168 (865-3306) | 2152 (1616-2361) | 1324 (924-1528) | 3466 (2681-5223) | 3708 (1484-6456) | 3264 (2708-4420) | 4152 (3272-5032) | 802 (563-1198) | 814 (464-2096) | 1684 (1316-1748) | 1568 |  |
| CSF NfH [pg/mL] | 928 (426-1662) | 656 (404-1133) | 1003 (735-1297) | 891 (488-1356) | 6145 (4132-8800) | 6693 (4298-10044) | 964 (684-1360) | 1116 (832-1392) | 1424 (792-2206) | 670 (368-1919) | 1820 (1562-1943) | 1064 (936-1208) | 2070 (1298-2488) | 1896 (1208-2024) | 808 (574-1688) | 698 (496-900) | 1162 (784-1471) | 1052 (744-2023) | 1964 (1232-2084) | 2332 |  |

**Table S2. Sex-disaggregated demographic data of the diagnostic cohort.**

*Sex information was obtained through self-report from study participants.

Data is reported as median (Interquartile range).

Abbreviations: AD, Alzheimer’s disease; ALS, amyotrophic lateral sclerosis; bvFTD, behavioural variant frontotemporal dementia; CTRL, non-neurodegenerative controls; CTRL.DD, control patients with initial diagnostic suspicion of ALS but finally diagnosed with another condition; CSF, cerebrospinal fluid; FTD, frontotemporal dementia; LBD, Lewy body dementia; lvPPA, logopenic variant primary progressive aphasia; NfH, neurofilament heavy chain; NfL, neurofilament light chain; NfM, neurofilament medium chain; nfvPPA, non-fluent variant primary progressive aphasia; PD, Parkinson’s disease; PDD, Parkinson’s disease dementia; svPPA, semantic variant primary progressive aphasia.


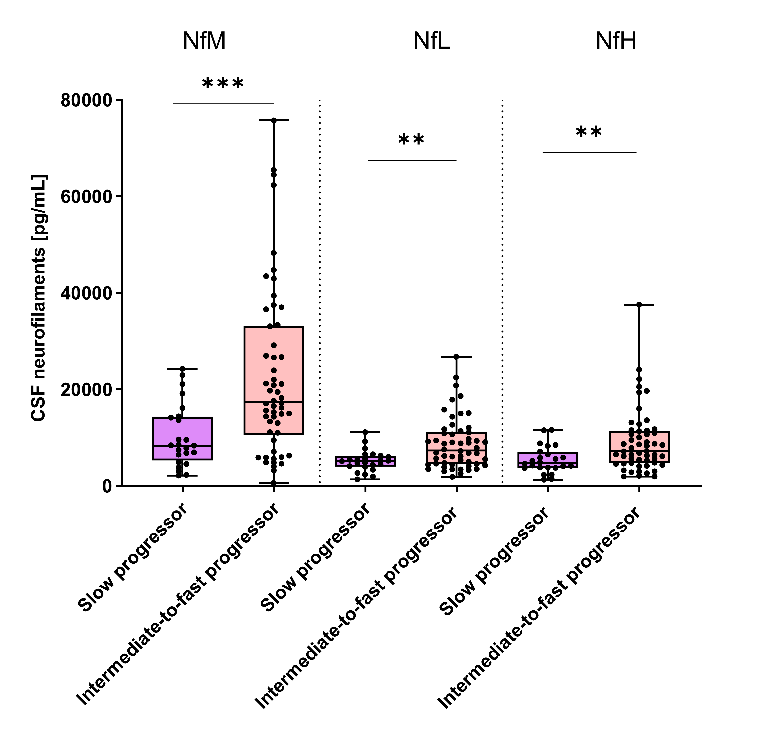


**Figure S3. CSF Neurofilament levels in patients with ALS stratified by disease progression rate.**

Patients with ALS were categorised into two groups based on their disease progression rate (ΔFS): slow progressors (ΔFS ≤ 0.4 points/month) and intermediate-to-fast progressors (ΔFS > 0.4 points/month), where ΔFS was calculated as (48 – ALSFRS-R at diagnosis) divided by the number of months from symptom onset to diagnosis. CSF concentrations of NfM, NfL, and NfH were compared between the two groups. NfM showed the most significant difference between groups (p = 0.0007), followed by NfL (p = 0.0035) and NfH (p = 0.0055), indicating their association with disease progression severity. Pairwise comparisons between groups were performed using the Mann–Whitney U test. Box plots display the median, interquartile range, and individual data points for each neurofilament.

|  | Slow progressors | Intermediate-to-fast progressors |
| --- | --- | --- |
| N | 24 | 54 |
| Age[year] | 62 (51-70) | 65 (55-74) |
| Female/Male | 13/11 | 25/29 |
| Progression rate | 0.21 (0.14 - 0.33) | 0.79 (0.55 - 1.26) |
| CSF NfM [pg/mL] | 8387 (5292-14354) | 17435 (10650-33139) |
| CSF NfL [pg/mL] | 5211 (4006-6248) | 7396 (4507-11186) |
| CSF NfH [pg/mL] | 4725 (3783-6943) | 7201 (4738-11401) |

**Table S3. Demographic data of the subgroup of patients with ALS stratified by progression rate.**


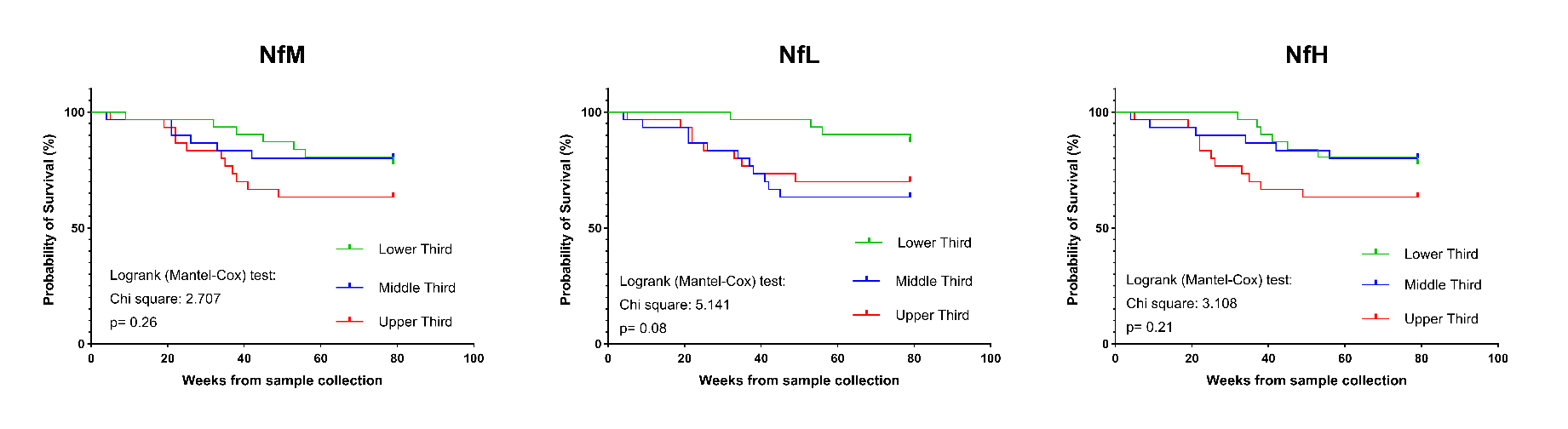


**Figure S4. Kaplan–Meier survival curves.**

The ALS cohort was stratified into tertiles according to neurofilament levels at the time of sample collection, and Kaplan–Meier survival curves were generated to depict survival probabilities over time. Log-rank (Mantel–Cox) test results are presented for each neurofilament subunit.

**Neurofilaments ratio in diagnostic groups**


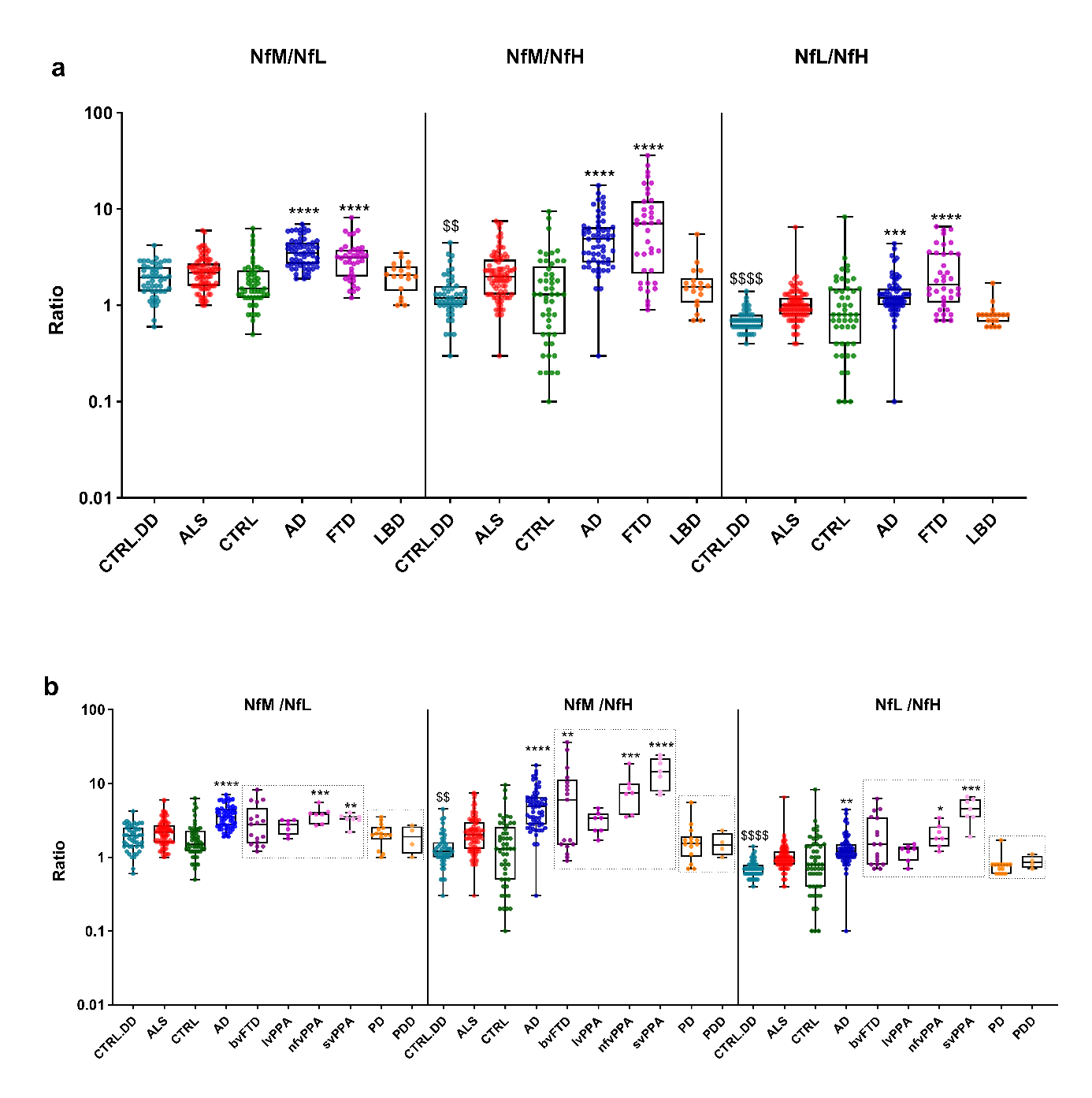
The calculation of neurofilament ratios revealed significantly higher values for all three ratios in the AD (NfM/NfL: p<0.0001; NfM/NfH: p<0.0001; NfL/NfH: p=0.0005) and FTD (NfM/NfL: p<0.0001; NfM/NfH: p<0.0001; NfL/NfH: p<0.0001) cohorts compared to the CTRL group. In contrast, the ALS cohort showed no significant differences from CTRL. However, the NfM/NfH (p=0.0029) and NfL/NfH (p<0.0001) ratios were significantly different in the ALS cohort compared to the CTRL.DD group (Figure S5a). Furthermore, pairwise comparisons using Mann-Whitney U test within FTD subgroups revealed significant lower levels in lvPPA compared to nfvPPA (NfM/NfL: p=0.0274; NfM/NfH: p=0.0064; NfL/NfH: p=0.0169), and to svPPA (NfM/NfL: p=0.0152; NfM/NfH: p=0.0006; NfL/NfH: p=0.0006). Additionally, svPPA cohort showed significant higher values compared to bvFTD (NfM/NfH: p=0.0385; NfL/NfH: p=0.0049) and to nfvPPA (NfL/NfH: p=0.0041). No significant differences were observed within the LBD subgroups (Figure S5b).

**Figure S5. Ratio of neurofilament subunits across different diagnostic groups.**


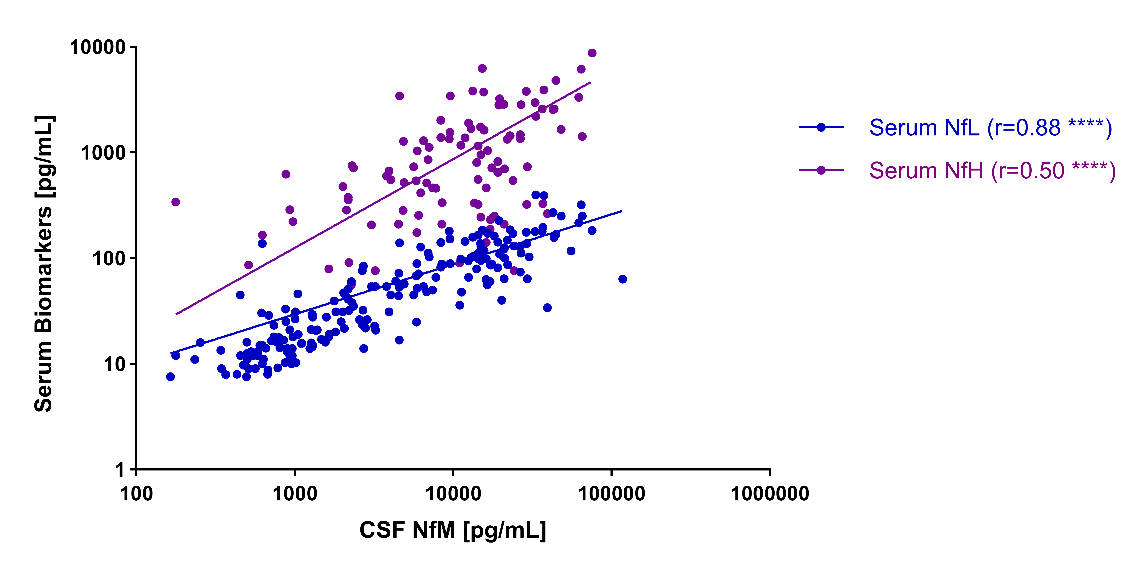
Ratios of CSF NfM, NfL and NfH concentrations in the diagnostic groups (**a**) and in the extended diagnostic groups (**b**). Statistically significant differences between the patient cohorts and CTRL are indicated with star symbols (*), while the comparison between patients with ALS and the CTRL.DD cohort is marked with dollar signs ($). Additional significant differences within the diagnostic cohorts are observed with the following p-values; NfM/NfL (ALS vs AD: p<0.0001; ALS vs FTD: p=0.0131; AD vs LBD: p<0.0001; FTD vs LBD: p=0.0485), NfM/NfH (ALS vs AD: p<0.0001; ALS vs FTD: p<0.0001; AD vs LBD: p<0.0001; FTD vs LBD: p<0.0001), and NfL/NfH (ALS vs AD: p=0.0153; ALS vs FTD: p=0.0001; AD vs LBD: p=0.0002; FTD vs LBD: p<0.0001). Further significant differences in extended subgroups are as following; NfM/NfL (AD vs PD: p=0.0038), NfM/NfH (ALS vs nfvPPA: p=0.0202; ALS vs svPPA: p=0.0010; AD vs PD: p=0.0003; nfvPPA vs PD: p=0.0061; svPPA vs PD: 0.0004; svPPA vs PDD: 0.0310), NfL/NfH (ALS vs svPPA: p=0.0019; AD vs PD: p=0.0013; bvFTD vs PD: p=0.0181; nfvPPA vs PD: p=0.0015; svPPA vs PD: p<0.0001). Displayed are the median concentration, the 25% and 75% percentiles and whiskers from minimum to maximum. Groups were compared by Kruskal-Wallis test and Dunns post hoc test.( * p< 0.05; ** p< 0.01; *** p< 0.001; **** p<0.0001; and $$$$ p<0.0001) AD, Alzheimer’s disease; ALS, amyotrophic lateral sclerosis; bvFTD, behavioural variant frontotemporal dementia; CTRL, non-neurodegenerative controls; CTRL.DD, control patients with initial diagnostic suspicion of ALS but finally diagnosed with another condition; CSF, cerebrospinal fluid; FTD, frontotemporal dementia; LBD, Lewy body dementia; lvPPA, logopenic variant primary progressive aphasia; NfH, neurofilament heavy chain; NfL, neurofilament light chain; NfM, neurofilament medium chain; nfvPPA, non-fluent variant primary progressive aphasia; PD, Parkinson’s disease; PDD, Parkinson’s disease dementia; svPPA, semantic variant primary progressive aphasia.

**Figure S6. Correlation between CSF NfM and serum NfL and NfH in a subgroup of patients with neurodegenerative diseases.**

Scatter plot shows the correlations between CSF NfM and serum NfL (shown in blue) and NfH (shown in purple) in a subgroup of patients with neurodegenerative diseases. Serum NfL values (n = 195) and NfH values (n = 100) were measured using Ella commercial immunoassays. A strong correlation was observed between CSF NfM and serum NfL (r = 0.8818, 95% CI: 0.8450–0.9104, p < 0.0001), and a moderate correlation was found between CSF NfM and serum NfH (r = 0.5029, 95% CI: 0.3348–0.6399, p < 0.0001). Correlation analysis was performed using Spearman’s correlation coefficient. Non-linear regression curves are shown for each biomarker.


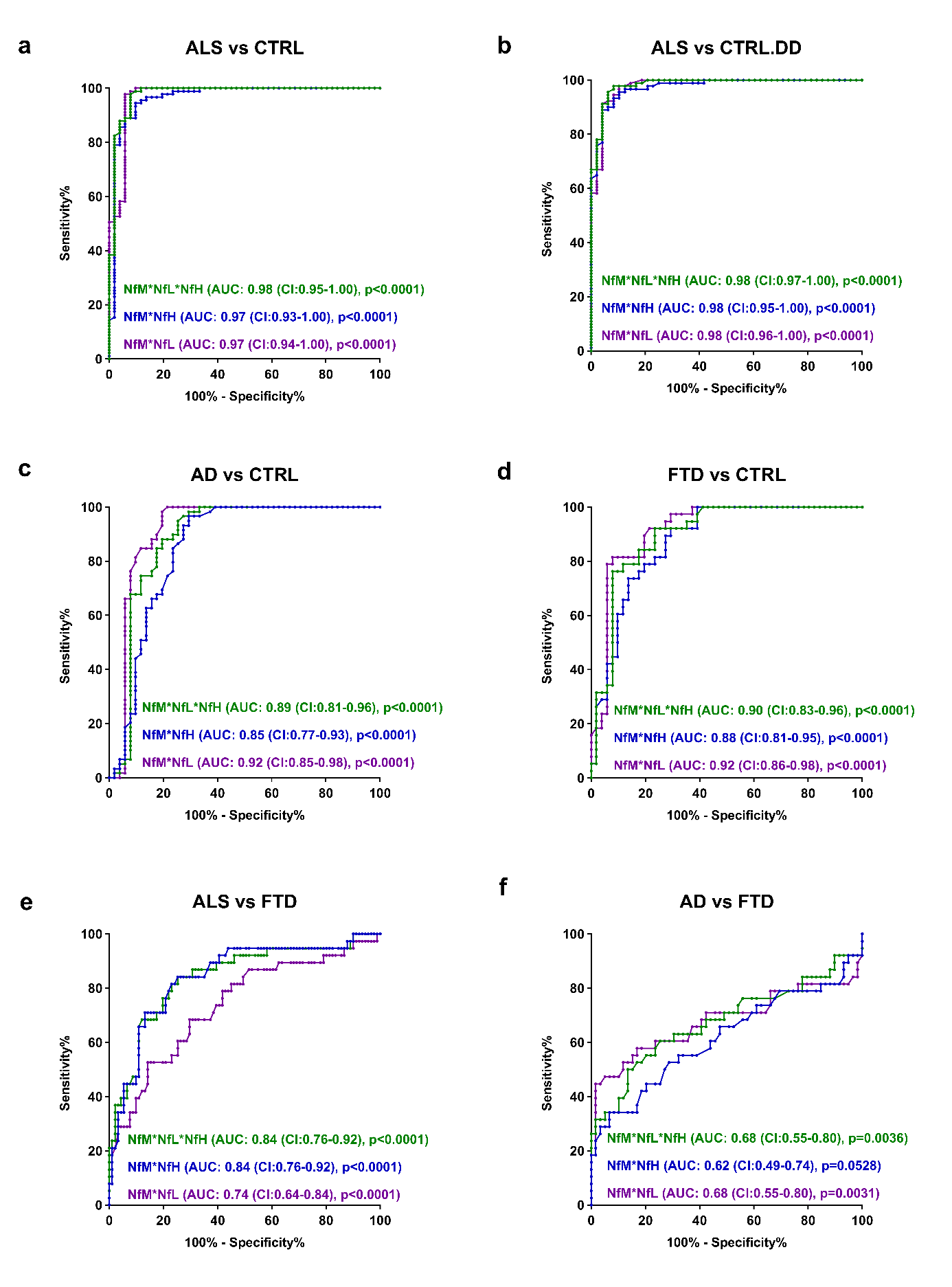


**Figure S7. ROC analysis of combined values of CSF neurofilament proteins.**

The panels show the results of the ROC analyses comparing the combination of neurofilament proteins in the CSF for discriminating the following groups: (**a**) ALS vs. CTRL, (**b**) ALS vs. CTRL.DD, (**c**) AD vs. CTRL, (**d**) FTD vs. CTRL, (**e**) ALS vs. FTD, and (**f**) AD vs. FTD. AD, Alzheimer’s disease; ALS, amyotrophic lateral sclerosis; CI, confidence intervals; CTRL, non-neurodegenerative controls; CTRL.DD, control patients with initial diagnostic suspicion of ALS but finally diagnosed with another condition; CSF, cerebrospinal fluid; FTD, frontotemporal dementia; NfH, neurofilament heavy chain; NfL, neurofilament light chain; NfM, neurofilament medium chain; NfL, neurofilament light chain protein; ROC, receiver operating characteristic.

| Biomarker | Cohort comparison | Cut-off [pg/mL] | Sensitivity% | 95% CI | Specificity% | 95% CI | Likelihood ratio | Youden Index |
| --- | --- | --- | --- | --- | --- | --- | --- | --- |
| CSF NfM | ALS vs CTRL | > 3531 | 90.11 | 82.26% to 94.71% | 94.12 | 84.08% to 98.40% | 15.32 | 0.8423 |
| CSF NfL | ALS vs CTRL | > 1314 | 100 | 95.95% to 100.0% | 91.84 | 80.81% to 96.78% | 12.25 | 0.9184 |
| CSF NfH | ALS vs CTRL | > 1915 | 96.7 | 90.75% to 99.10% | 86.27 | 74.28% to 93.19% | 7.046 | 0.8297 |
| CSF NfM | ALS vs CTRL.DD | > 4595 | 86.81 | 78.35% to 92.29% | 95.83 | 86.02% to 99.26% | 20.84 | 0.8264 |
| CSF NfL | ALS vs CTRL.DD | > 1741 | 97.8 | 92.34% to 99.61% | 95.83 | 86.02% to 99.26% | 23.47 | 0.9363 |
| CSF NfH | ALS vs CTRL.DD | > 1848 | 96.7 | 90.75% to 99.10% | 93.75 | 83.16% to 97.85% | 15.47 | 0.9045 |
| CSF NfM | AD vs CTRL | > 1867 | 100 | 93.89% to 100.0% | 82.35 | 69.75% to 90.43% | 5.667 | 0.8235 |
| CSF NfL | AD vs CTRL | > 885.5 | 89.83 | 79.54% to 95.26% | 83.67 | 70.96% to 91.49% | 5.502 | 0.735 |
| CSF NfH | AD vs CTRL | > 672.0 | 91.53 | 81.65% to 96.33% | 45.1 | 32.27% to 58.62% | 1.667 | 0.3663 |
| CSF NfM | FTD vs CTRL | > 1347 | 92.11 | 79.20% to 97.28% | 76.47 | 63.24% to 86.00% | 3.914 | 0.6858 |
| CSF NfL | FTD vs CTRL | > 893.5 | 92.11 | 79.20% to 97.28% | 83.67 | 70.96% to 91.49% | 5.641 | 0.7578 |
| CSF NfH | FTD vs CTRL | > 696.0 | 89.47 | 75.87% to 95.83% | 47.06 | 34.05% to 60.48% | 1.69 | 0.3653 |
| CSF NfM | ALS vs FTD | < 10947 | 68.42 | 52.54% to 80.92% | 61.54 | 51.27% to 70.87% | 1.779 | 0.2996 |
| CSF NfL | ALS vs FTD | < 3828 | 81.58 | 66.58% to 90.78% | 79.12 | 69.68% to 86.21% | 3.907 | 0.607 |
| CSF NfH | ALS vs FTD | < 2618 | 94.74 | 82.71% to 99.06% | 89.01 | 80.94% to 93.92% | 8.621 | 0.8375 |
| CSF NfM | AD vs FTD | > 8649 | 47.37 | 32.48% to 62.74% | 88.14 | 77.48% to 94.13% | 3.992 | 0.3551 |
| CSF NfL | AD vs FTD | > 2192 | 57.89 | 42.19% to 72.15% | 93.22 | 83.82% to 97.33% | 8.539 | 0.5111 |
| CSF NfH | AD vs FTD | > 1414 | 50 | 34.85% to 65.15% | 83.05 | 71.54% to 90.52% | 2.95 | 0.3305 |

**Table S4. Sensitivity, Specificity, and Youden Index Summary**

AD, Alzheimer’s disease; ALS, amyotrophic lateral sclerosis CTRL, non-neurodegenerative controls; CTRL.DD, control patients with initial diagnostic suspicion of ALS but finally diagnosed with another condition; CSF, cerebrospinal fluid; FTD, frontotemporal dementia; NfH, neurofilament heavy chain; NfL, neurofilament light chain; NfM, neurofilament medium chain.
